# Diagnostic Accuracy of CT Texture Analysis in Adrenal Masses: A Systematic Review

**DOI:** 10.3390/ijms23020637

**Published:** 2022-01-07

**Authors:** Filippo Crimì, Emilio Quaia, Giulio Cabrelle, Chiara Zanon, Alessia Pepe, Daniela Regazzo, Irene Tizianel, Carla Scaroni, Filippo Ceccato

**Affiliations:** 1 Department of Medicine DIMED, University of Padova, 35128 Padua, Italy; filippo.crimi@unipd.it (F.C.); emilio.quaia@unipd.it (E.Q.); giulio.cabrelle@gmail.com (G.C.); zanon.chiara.9@gmail.com (C.Z.); alessia.pepe@unipd.it (A.P.); daniela.regazzo@unipd.it (D.R.); irene.tizianel@gmail.com (I.T.); carla.scaroni@unipd.it (C.S.); 2 Institute of Radiology, University-Hospital of Padova, 35128 Padua, Italy; 3 Endocrine Disease Unit, University-Hospital of Padova, 35128 Padua, Italy

**Keywords:** adrenal glands, computed tomography, texture analysis, radiomics

## Abstract

Adrenal incidentalomas (AIs) are incidentally discovered adrenal neoplasms. Overt endocrine secretion (glucocorticoids, mineralocorticoids, and catecholamines) and malignancy (primary or metastatic disease) are assessed at baseline evaluation. Size, lipid content, and washout characterise benign AIs (respectively, <4 cm, <10 Hounsfield unit, and rapid release); nonetheless, 30% of adrenal lesions are not correctly indicated. Recently, image-based texture analysis from computed tomography (CT) may be useful to assess the behaviour of indeterminate adrenal lesions. We performed a systematic review to provide the state-of-the-art of texture analysis in patients with AI. We considered 9 papers (from 70 selected), with a median of 125 patients (range 20–356). Histological confirmation was the most used criteria to differentiate benign from the malignant adrenal mass. Unenhanced or contrast-enhanced data were available in all papers; TexRAD and PyRadiomics were the most used software. Four papers analysed the whole volume, and five considered a region of interest. Different texture features were reported, considering first- and second-order statistics. The pooled median area under the ROC curve in all studies was 0.85, depicting a high diagnostic accuracy, up to 93% in differentiating adrenal adenoma from adrenocortical carcinomas. Despite heterogeneous methodology, texture analysis is a promising diagnostic tool in the first assessment of patients with adrenal lesions.

## 1. Introduction

Adrenal incidentalomas (AIs) are adrenal neoplasms discovered during a procedure not performed for suspected adrenal disease [1,2]. Their prevalence is age dependent (up to 10–15% in adults >60–70 years old), and their detection is increased in recent years, because of the large availability of imaging medical equipment such as computed tomography (CT) and magnetic resonance (MR), resulting in more than 200 CT and 100 MR scans for 1000 people [3].

Overt endocrine secretion (glucocorticoids, mineralocorticoids, and catecholamines, characterising, respectively, Cushing’s syndrome, primary aldosteronism, and pheochromocytoma) should be ruled out in all patients with AI by the measurement of serum cortisol after 1 mg dexamethasone (the overnight suppression test), aldosterone-to-renin ratio, and plasma or urinary metanephrines [2,4,5]. Nevertheless, the majority of them are non-secreting cortical adenomas [1,2].

At the first evaluation, it is also recommended to rule out malignancy [2]. Malignant AIs could be a primary adrenal disease (adrenocortical cancer, ACC, or malignant pheochromocytoma), or metastases, whose prevalence in AIs is 7.5%. A medical history positive for extra-adrenal cancer or imaging prescription for active cancer are common: Metastatic AI was 22 times more likely during cancer staging [6], and 27% of malignant tumours (especially lung, breast, gastric, liver, and pancreatic cancer) cause adrenal metastasis on autopsy studies [7]. On the contrary, adrenal metastases could be excluded in 96% of AIs by multidisciplinary evaluation in patients affected by colorectal cancer and AIs (10.5% in 500 patients) [8].

The first radiological approach should discriminate benign adenomas from malignant lesions: An attenuation value <10 Hounsfield unit (HU) in unenhanced computed tomography is able to diagnose lipid-rich benign cortical adenomas [2,9]. From a functional point of view, steroid-secreting cells in the adrenal cortex contain a large number of lipids (steroidogenesis comes from cholesterol); therefore, non-functioning benign adrenal adenomas usually contain a high number of lipids [10], recognised by unenhanced CT and quantified with the attenuation value. On the other hand, 30% of benign adrenal adenomas present an attenuation value of >10 HU (lipid-poor adenomas). During contrast-enhanced CT, adenomas typically enhance quickly and show prompt washout, malignant adrenal lesions usually enhance, but they have slower washout. A relative washout (portal HU–delayed HU/portal HU) >40% or an absolute washout (portal HU–delayed HU/portal HU–unenhanced HU) >60% after 15 min indicate a benign lesion [11].

These approaches are not able to characterise all adrenal masses, and adrenal biopsy has a limited role [12]. Large adrenal masses are often heterogeneous for the presence of necrosis, haemorrhage, calcifications, and intracellular lipid content; therefore, neither CT nor MR scans are able to define their nature. Recently, an emerging field of radiology, called radiomics, uses image-based texture analysis from CT and MR to provide quantitative parameters that may be useful to measure the heterogeneity of tumours [13,14].

The aim of the present review is to provide an up-to-date, state-of-the-art application of texture analysis in patients with AI.

## 2. Results

All the studies identified by the literature search were observational retrospective, and their setting was a university hospital. According to selection criteria, nine papers were considered in this systematic review, reported in Table 1. Their QUADAS-2 is reported in Table 2.

The median number of patients included in the studies was 125 (range 20–356).

In six studies, histopathological examination was used as a gold standard reference to confirm the malignant or benign nature of the adrenal tumours [15,16,17,18,19,20]. Two studies used a combination of histopathological and follow-up data [21,22]. In one study, the gold standard employed to discriminate between benign versus malignant lesions was unclear [23].

Six studies included adrenal metastases among malignant adrenal lesions [15,16,18,19,20,22,23]. Two studies focused the analysis on the differential diagnosis between adrenal cortisol lesions (ACC and adenomas) [17,21].

Notably, two studies [15,23] included pheochromocytomas, which are usually diagnosed by serum or urine fractionated metanephrines instead of imaging.

Three studies included only CT scans after injection of intravenous contrast medium [16,18,20], while the others reported the employment of both unenhanced and contrast-enhanced CT images for texture analysis.

The most frequently used software for texture analysis was TexRAD (n = 3) [15,16,18], followed by PyRadiomics (n = 2) [17,20].

Four papers analysed the whole volume of the adrenal lesions [17,21,22,23], while five focused only on a single region of interest (ROI) drawn in an axial slice [15,16,18,19,20].

In five studies, the ROIs of the adrenal lesions were drawn by two radiologists in consensus [15,17,18,20,21], while in three other studies, only one radiologist was involved [16,19,22] Fare clic o toccare qui per immettere il testo., and in one study the number of investigators was not specified [23].

Three groups collected textural features applying an image filtration technique with a Laplacian of Gaussian spatial bandpass filter that used different spatial scaling filters (SSF), in order to enhance features of different sizes and intensity variation in the adrenal lesions [15,16,18].

Different texture features were reported to be significantly different between benign and malignant adrenal lesions (first- and second-order parameters). First-order statistics describe the properties of individual pixels, while second-order statistics also consider the spatial interdependency or co-occurrence of two pixels at their specific positions.

All studies but two [19,21], drew receiver-operating characteristic (ROC) curves to calculate the accuracy of the textural parameters in discriminating benign from malignant lesions. The pooled median area under the ROC curve (AUC) was 0.85 (range 0.67–0.89).

In one study [23], the authors focused on the accuracy of CT texture analysis in differentiating secreting from non-secreting masses (the AUC was 0.93) and calcified versus non-calcified lesions (AUC was 1.00).

Notably, four studies found the mean attenuation value at unenhanced CT scan among the parameters that showed a statistically significant difference between the benign and malignant adrenal masses; one reported a higher mean densitometry of the benign lesion [15] while the other three showed a higher mean densitometry of the malignant lesions [17,21,22].

**Figure 1:**
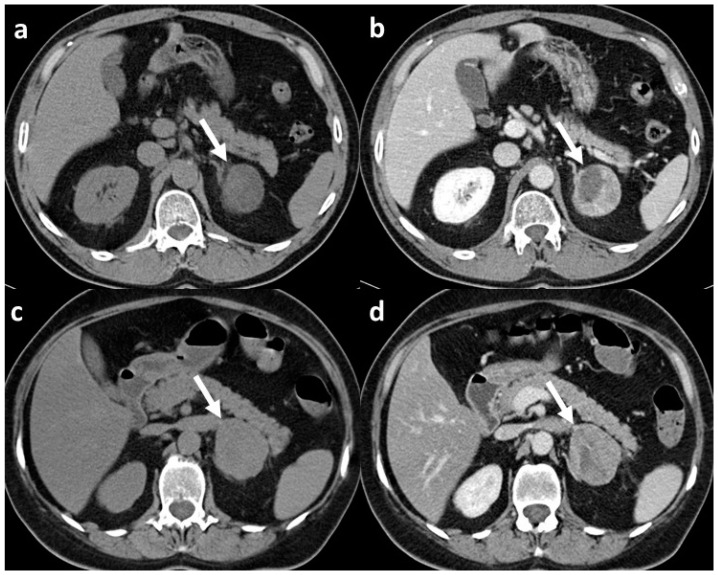
CT scans of adrenal adenoma and adrenocortical carcinoma both with CT features suspect for malignant lesion: (**a**) unenhanced scan of histopathologically confirmed adrenal adenoma (arrow) with mean densitometry of 22 HU; (**b**) venous phase scan of the same adrenal adenoma (arrow), at late scan (not shown) the relative washout of contrast medium was <40%; (**c**) unenhanced scan of histopathologically confirmed adrenocortical carcinoma (arrow) with mean densitometry of 28 HU; (**d**) venous phase scan of the same adrenocortical carcinoma (arrow), and also in this case, at late scan (not shown), the relative washout of contrast medium was <40%.

**Figure 2:**
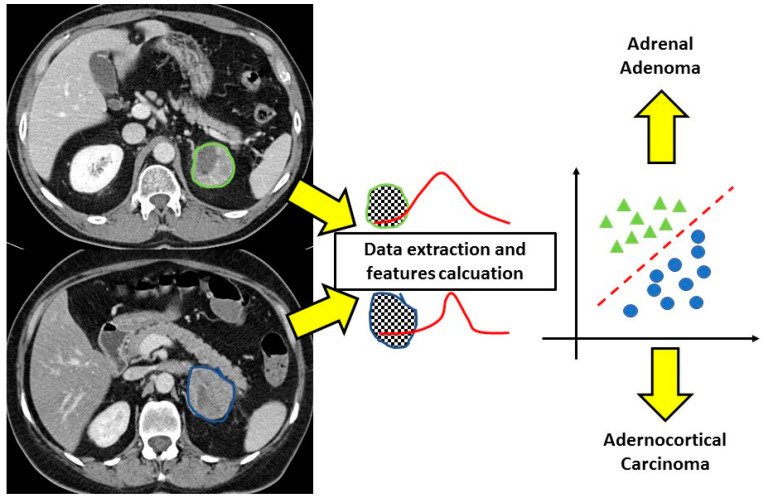
CT texture analysis of adrenal masses. After the contouring of the lesions, the software extracts different features from the regions of interest allowing correct classification of the adenoma and adrenocortical carcinoma that at conventional imaging would not have been distinguished.

Focusing on the two studies that compared adrenal adenoma and adrenocortical carcinomas, the results showed a very good performance of the CT texture analysis in differentiating benign from malignant lesions, with an AUC of 0.86 reported by Elmohr et al. [17] and an accuracy of 93% reported by Torresan et al. [21] (Figure 1 and Figure 2).

## 3. Discussion

During clinical practice, several physicians are facing a large and increasing number of patients with AIs, due to the availability of imaging facilities (especially CT and MR) [3]. At the first evaluation, it is important to determine the non-secreting and benign behaviour of the adenoma. In the case of the non-conclusive first approach, a multidisciplinary evaluation is suggested, in order to personalise the approach with nuclear/functional imaging or second-level dynamic endocrine tests [24].

A novel and emerging approach to characterise the behaviour of adrenal lesions is the measurement of steroid precursors using mass spectrometry, which allows the identification of several steroids and intermediate patterns [25]. Malignant lesions have a specific steroid fingerprint, secondary to immature steroidogenesis. Despite a large interindividual heterogeneity, a ‘steroidomic’ approach is able to identify ACC and differentiate it from adenomas [26], achieving in selected cases >90% sensitivity and specificity [25]. Some steroid precursors/metabolites (especially tetrahydro-11-deoxycortisol and 17-hydroxypregnenolone) are peculiar in the setting of adrenocortical malignancy [25]; their combination with tumour size and lipid content is able to predict adenoma behaviour [27]. One of the drawbacks of such a steroidomic approach is its limited availability (only in selected academic centres with mass spectrometry facilities) and time-consuming analysis.

According to several guidelines and recent papers [1,2,28,29,30], small size and a high lipid content (<4 cm in size and <10 HU attenuation value) are accepted as markers of a benign lesion. Nonetheless, up to 30% of AIs do not fulfil the well-established criteria of a benign lesion, and novel approaches are needed. Recently, image-based texture analysis from CT and MR provides quantitative parameters that may be useful to measure the presence of necrosis, haemorrhage, calcifications, and intracellular lipid content, allowing to differentiate benign from malignant tumours [13,14,31,32,33].

From 2018 to 2021, several papers regarding texture analyses in patients with adrenal lesions have been published. After our systematic review, only nine works presented an adequate level of evidence and were analysed. Unfortunately, the reported data were inhomogeneous to such an extent that a meta-analysis was not computable because true/false and positive/negative cases were not reported in an adequate number of studies.

Our results show that texture analysis has a good accuracy in differentiating benign from malignant adrenal lesions (pooled AUC 0.85); moreover, it performed even better in the differentiation between cortical lesions (adenomas and ACC). It is not a minor concern, because cortisol secretion can be overt or subclinical in an adenoma or a carcinoma; therefore, an endocrine differentiation of cortical masses is not always feasible.

Some groups focused on the identification of secreting adrenal lesions and included pheochromocytomas among the cases analysed. However, in clinical practice, when a physician is facing the question of whether the lesion is benign or not, the study of the endocrine function of a lesion is a pivotal tool. As a matter of fact, the adrenal cortex is able to synthetise and secrete steroids, and the adrenal medulla is deputed to the production of catecholamines, and biochemical tests are very accurate in identifying these hormonal products. Therefore, the study of the endocrine secretion must proceed close to radiological evaluations, and the use of texture analysis for hormonal production seems to be a time-consuming application due to the wide availability of very accurate laboratory examinations. Among the nine papers selected for the systematic review, only two were able to reply to a research question that can fulfil the population–intervention–comparison–outcome (PICO) model [34]. In the authors’ opinion, an answer to the proper research question of texture analysis addressing ‘the distinction between adrenal cortical adenoma and carcinoma’ can be found in the studies by Elmohr et al. [17] and Torresan et al. [21].

Notably, different studies underlined the good performance of CT texture analysis performed in the unenhanced scan. These data are of much interest mainly for two reasons: the first is that they highlight the application of texture analysis also in the field of the newly discovered AIs since many times the examination performed is an unenhanced CT scan performed for other reasons than a suspected adrenal pathology, while the second reason is that in the future, the characterisation of the adrenal masses with texture analysis will spare the contrast medium injection in these patients, allowing a reduced risk of allergic reaction and a negative impact on renal function [35,36].

Radiomics and, in particular, CT texture analysis can represent a more accessible and less time-consuming tool to differentiate benign from malignant adrenal lesions, compared with steroidomic approaches. Almost all centres can afford to perform a CT examination on a patient with AI, and many different software programs are available to extract textural features from CT images. The easy applicability of this new technique brought different study groups to test the efficacy and accuracy of texture analysis in adrenal lesions. Since the expanding use of radiomic in this context, the aim of the present review is to summarise the body of evidence in the literature in order to provide a guide for future application of this method of analysis.

Only selected studies considered a histological confirmation of the adrenal mass. This is a critical matter because, in clinical practice, the adrenal can be the site of a large series of primary and secondary, either benign or malignant, diseases [37]. Therefore, adrenalectomy is of utmost importance, at least to obtain the confirmation of the adenoma. In clinical practice, the use of adrenal biopsy is limited to selected cases, especially in patients with a history of extra-adrenal malignancy [12]. Lastly, we must consider that the results we are facing are not all derived from the same CT scanner, and it has been reported that different types of images acquisition can increase the variability in the densitometry value of each pixel or voxel, affecting the reproducibility of the results obtained by each study and, therefore, the usefulness of texture analysis [38].

The 18-fluoro-2-deoxy-D-glucose (18F-FDG) positron emission tomography (PET), alone or combined with CT or MR, could be useful to characterise malignant forms. An adrenal mass is likely malignant when the uptake of 18-FDG is higher than that of the liver. However, false positives (sarcoidosis, tuberculosis, lipid-poor or cortisol-secreting adenomas, and pheochromocytomas) or negatives (haemorrhage or necrosis are common in malignant lesions) must be considered [39,40,41,42,43]. Finally, PET is considered second-line imaging because it is performed in selected cases in a limited number of centres; on the contrary, CT is widely available (therefore, an effort for texture analysis in CT can produce a significant result).

There are several limitations. Firstly, the low number of studies published in the literature and the huge variety of techniques and parameters used to test the accuracy of texture did not allow us to perform a meta-analysis. However, despite its limitations, the pooled AUC can be a good indicator of the performance of this new radiological technique. Secondly, the histopathological diagnoses of benign and malignant lesions varied consistently, and such heterogeneity could have affected the results. Finally, the CT scanning protocols were different among the studies included in the review and, therefore, is a source of variability that can affect the comparison of the results.

In conclusion, from the data collected in this systematic review, texture analysis appears as a new promising diagnostic tool in the adrenal tumoral pathologies; nevertheless, further prospective multicentric studies are needed to confirm its role in the clinical setting.

## 4. Materials and Methods

### 4.1. Search Strategy

We used three search engines (PubMed, Web of Science, and Scopus), from inception through to July 2021, for a literature search based on the PRISMA criteria [44]. We used the following terms and their variants in the title, abstract and keyword fields, and MeSH fields where available, adapting the search syntax where necessary: ‘adrenal AND computed tomography-CT-AND texture analysis; adrenal AND computed tomography-CT-AND texture analysis AND radiomics’.

The references we included in the review were also searched manually for papers not identified by the initial literature search.

Exclusion criteria were the following: (I) articles not written in English; (II) studies containing aggregated data or data duplicated from previously published works; (III) review articles; (IV) letters; (V) case reports; (VI) editorials. No restrictions were placed on study design or population.

### 4.2. Review Protocol and Data Extraction

Three authors (G.C., C.Z., and F.Cr.) independently screened all titles and abstracts generated by analysing the databases. Afterwards, the same authors screened the full texts of all the relevant papers identified according to the inclusion criteria. Any disagreement regarding article suitability for the inclusion in the review was fixed by discussion or, failing this, by referral to a senior author (F.Ce.).

**Figure 3:**
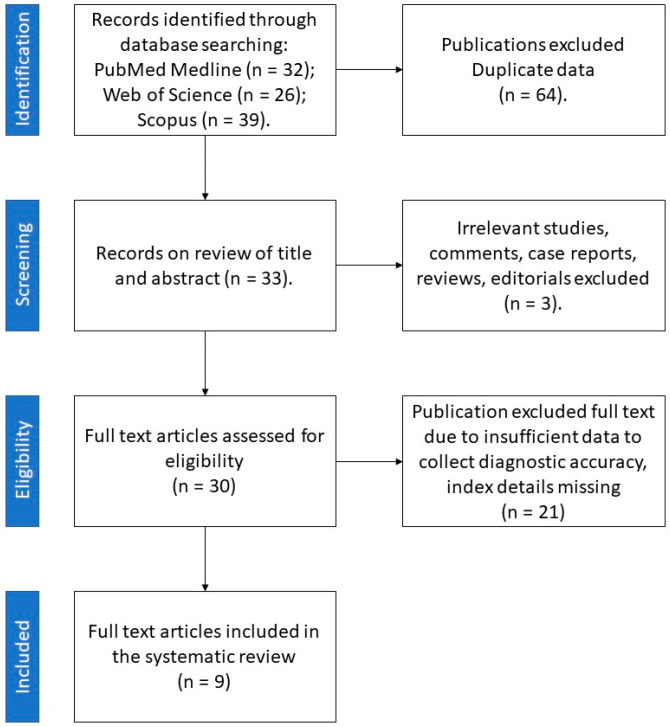
Retrieval flowchart to obtain study data for systematic review.

Among 70 papers obtained from the literature search, 9 reports published between 2018 and 2021 met the inclusion criteria (Figure 3).

Data from these studies were extracted using a standardised pro forma in Microsoft Excel (Redmond, WA, USA). The information extracted from each study included author, year of publication, study design, number of cases, and main findings.

All the studies were scored based on the Quality Assessment of Diagnostic Accuracy Studies (QUADAS) version 2 [45]. Discrete variables were expressed as means ± standard deviations, or medians and interquartile ranges (IQR), as appropriate. Categorical data were described as absolute numbers and percentages.

## Figures and Tables

**Table 1:** Characteristics of the papers selected for the systematic review.

First Author	Year of Publication	Study Type	Number of Patients	Type of Adrenal Lesions	Unenhanced CT/Contrast-Enhanced CT	Number of Radiologists Involved	Observers Blinded to Clinical/Histopathological Data	Reference Standard	Texture Analysis Software	Spatial Scaling Factors	Number of Textural Features Selected (Unenhanced CT/Contrast-Enhanced CT)	Pooled AUC of the Study (Benign vs. Malignant Lesion)
Shi B	2018	Observational retrospective	225	Lipid poor adenomas, metastases, pheochromocytoma	+/+	2	Yes	Histology	TexRAD	0, 2, 3, 4, 5, 6	5/4	0.81
Yu HS	2020	Observational retrospective	125	Benign (not specified), metastases	−/+	1	Yes	Histology	TexRAD	0, 2, 3, 4, 5, 6	0/36	0.89
Elmohr MM	2019	Observational retrospective	54	Adenomas, adrenocortical carcinomas	+/+	2	Yes	Histology	PyRadiomics	NA	3/2	0.86
Ho LM	2019	Observational retrospective	20	Lipid poor adenomas, metastases, adrenocortical carcinomas	+/+	1	NA	Histology or radiological features (rapid size increase)	Lesion Tool	NA	9/18	0.85
Shoemaker K	2018	Observational retrospective	356	Adrenocortical carcinomas, adenomas, benign (not specified), haemorrhages, adrenal hyperplasia, lymphoma, malignant (not specified), metastases, myelolipomas, neurogenic tumours, pheochromocytomas	NA	NA	NA	NA	C++ program	NA	37	0.78 0.93 (functioning vs. non-functioning) 1 (calcified vs. non-calcified)
Andersen MB	2021	Observational retrospective	160	Benign (not specified), metastases of lung tumours (adenocarcinoma, squamous cell carcinoma, neuroendocrine tumours, unclassified NSCLC or SCLC)	−/+	2	Yes	Histology	TexRAD	NA	0/25	0.67
Li X	2021	Observational retrospective	204	Benign (not specified), malignant (not specified)	+/+	1	NA	Histology	HRGSDP method	NA	5/5	NA
Moawad AW	2021	Observational retrospective	40	Benign (not specified), metastases, adrenocortical carcinomas	+/+	2	NA	Histology	PyRadiomics	NA	1/3	0.85
Torresan F	2021	Observational retrospective	30	Adenomas, benign incidentalomas, adrenocortical carcinomas	+/+	2	Yes	Histology or follow-up	PMOD	NA	16/24	NA

+: performed; −: not performed.

**Table 2:** Quality Assessment of Diagnostic Accuracy Studies (QUADAS) version 2 evaluation of the papers selected for the systematic review.

					Risk of Bias				Applicability Concern		
First Author	Year	Setting	Type of Study	Design	Patient Selection	Index Test	Reference Standard	Flow and Timing	Patient Selection	Index Test	Reference Standard
Shoemaker	2018	University hospital	Observational	Retrospective	Unclear	Unclear	Low	Unclear	High	Low	Low
Elmohr	2019	University hospital	Observational	Retrospective	Low	Low	Low	Low	Low	Low	Low
Ho	2019	University hospital	Observational	Retrospective	Low	Low	High	Low	Low	Low	High
Shi	2019	University hospital	Observational	Retrospective	Low	Low	Low	Low	Low	Low	Low
Yu	2020	University hospital	Observational	Retrospective	Low	Low	Low	Low	Low	Low	Low
Andersen	2021	University hospital	Observational	Retrospective	Low	Low	Low	Low	Low	Low	Low
Li	2021	University hospital	Observational	Retrospective	Low	Unclear	Low	Low	Low	Unclear	Low
Moawad	2021	University hospital	Observational	Retrospective	Low	Low	Low	Low	Low	Low	Low
Torresan	2021	University hospital	Observational	Retrospective	Low	Low	Low	Low	Low	Low	Low

## Data Availability

All data are available in the manuscript.
